# A Robust Reweighted *L*_1_-Minimization Imaging Algorithm for Passive Millimeter Wave SAIR in Near Field

**DOI:** 10.3390/s151024945

**Published:** 2015-09-25

**Authors:** Yilong Zhang, Yuehua Li, Shujin Zhu, Yuanjiang Li

**Affiliations:** 1 School of Electronic and Optical Engineering, Nanjing University of Science and Technology, Nanjing 210094, China; E-Mails: 311040779@njust.edu.cn (Y.Z.); zsj0577@163.com (S.Z.); liyuanjiang@just.edu.cn (Y.L.); 2 Institute of Electronic and Information, Jiangsu University of Science and Technology, Zhenjiang 212000, China

**Keywords:** passive millimeter wave, SAIR, reweighted *L*_1_-minimization, prior information

## Abstract

The Compressive Sensing (CS) approach has proven to be useful for Synthetic Aperture Interferometric Radiometer (SAIR) imaging because it provides the same high-resolution capability while using part of interferometric observations compared to traditional methods using the entirety. However, it cannot always obtain the sparsest solution and may yield outliers with the non-adaptive random measurement matrix adopted by current CS models. To solve those problems, this paper proposes a robust reweighted *L*_1_-minimization imaging algorithm, called RRIA, to reconstruct images accurately by combining the sparsity and prior information of SAIR images in near field. RRIA employs iterative reweighted *L*_1_-minimization to enhance the sparsity to reconstruct SAIR images by computing a new weight factor in each iteration according to the previous SAIR images. Prior information estimated by the energy functional of SAIR images is introduced to RRIA as an additional constraint condition to make the algorithm more robust for different complex scenes. Compared to the current basic CS approach, our simulation results indicate that RRIA can achieve better recovery with the same amount of interferometric observations. Experimental results of different scenes demonstrate the validity and robustness of RRIA.

## 1. Introduction

Synthetic Aperture Interferometric Radiometer (SAIR) technology represents a promising new approach to passive millimeter wave imaging for high-resolution observations of the target scene, both in near field and far field, which can be applied to areas such as indoor security, aircraft navigation, environment monitoring, atmosphere monitoring and so on [1,2,3]. Based on coherent signal processing, SAIR measures the correlation between pairs of various nondirective antennas to achieve a larger aperture antenna, realizing high-resolution.

Caused by the fact that the multiplicative errors are dependent on the orientation, the reconstruction problem of SAIR imaging needs complex calculations and is ill-posed, which the traditional FFT and G matrix inversion method of SAIR cannot solve very well. To reduce the amount of data processing and achieve high-resolution, Compressive Sensing (CS) theory [4] is applied to SAIR based on the assumption that SAIR images can be sparsely represented in some spaces [5,6]. The CS approach achieves high-resolution with very limited correlation observations and its advantages are of great practical use because the signal-to-noise of SAIR images is improved by reducing the multiplicative errors in correlation observations [7]. The optimal sparse reconstruction algorithm is *L*_0_-minimization which needs a combinatorial search, thus it is NP-hard in general [8]. CS translates this problem into something more tractable by replacing the *L*_0_ norm with its convex relaxation *L*_1_ norm by obeying the Restricted Isometry Property (RIP). Ideally, CS can directly extract sparse solutions from correlation observations by solving a convex optimization. In practice, however, outliers are often present because the basic CS approach adopts a non-adaptive random measurement matrix, and the constant basis matrix for SAIR images and correlation observations of SAIR are nondirective and redundant [9,10]. Thus, the solution obtained by the basic CS approach is not sparse and robust enough.

In this paper, a robust reweighted *L*_1_-minimization imaging algorithm, called RRIA, is introduced to passive millimeter wave SAIR images in near field. The work is inspired by [11], which establishes a more valid optimizing rule that is similar to *L*_0_-minimization by iteratively solving a sequence of weighted *L*_1_-minimization problems. Specifically, RRIA adopts a new weight factor for SAIR imaging. The premise of the work is the assumption derived from SAIR image statistics that the RRIA model holds RIP which has been proved efficient by CS-based SAIR imaging. To further use SAIR images statistics, with the reweighted *L*_1_ model, RRIA employs prior information estimated by the energy functionals of SAIR images as a constraint condition to make the algorithm more robust [12,13]. The experimental results demonstrate that the RRIA enhances the sparsity and reduces anomalous values of recovered signals significantly compared with unweighted *L*_1_-minimization.

The rest of the paper is organized as follows: Section 2 gives a brief review of the SAIR model and establishes a new accurate description for near field SAIR imaging. The basic CS approach of SAIR is presented in Section 3 and the RRIA is described in Section 4. Experimental results to demonstrate the validity of the RRIA are presented in Section 5. Finally, Section 6 provides our conclusions.

## 2. Model of Passive Millimeter Wave SAIR Imaging in Near Field

**Figure 1:**
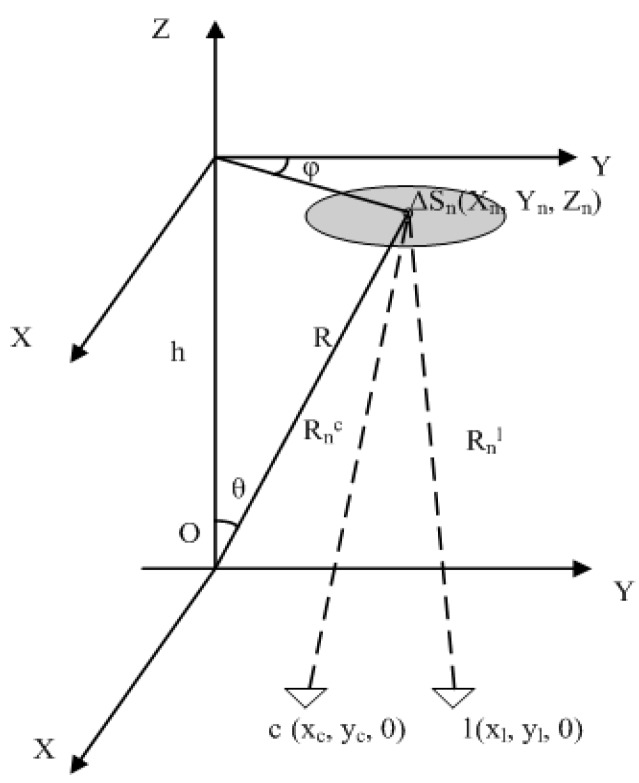
Interference measurement schematic.

Passive millimeter wave SAIR is a passive interferometry technique to obtain brightness temperature images by measuring the objects’ natural radiation in the millimeter wave band. It measures the correlation value directly, namely the visibility function [14], between pairs of spatially separated antennas receiving the electromagnetic signals from objects. Then SAIR reconstructs object brightness temperature images from the visibility function using algorithm like the FFT and G matrix inversion method. The binary interferometer is the basic unit of SAIR [15,16]. The geometric relationship of interferometry is presented in Figure 1.

A radiation source S is on the plane z = h while two antennas, labeled *c* and *l*, are located on the plane z = 0. Assuming that the radiation source *S* is dispersed into *N* small parts $\Delta S_{n}$, the visibility function is:

(1) $$V_{c,l}=\sum_{n=1}^{N}\frac{\sqrt{D_{1}D_{2}}}{4\pi }T(x_{n},y_{n})F_{c}(x_{n},y_{n})F_{l}^{*}(x_{n},y_{n})e^{-jK(R_{n}^{c}-R_{n}^{l})}\Delta S_{n}$$

where D is the antenna directivity, $T(x_{n},y_{n})$ is the source brightness temperature, $F_{c}(x_{n},y_{n})$ and $F_{l}(x_{n},y_{n})$ are the normalized antenna pattern of antenna *c* and *l*, respectively. $R_{n}^{c}$ and $R_{n}^{l}$ are the distances between the point source and interferometer antenna. *K* is circular wavenumber, defined as 2π/λ, where λ is the center wavelengths of the electromagnetic radiation received by SAIR imaging system. For the T- and U-shaped arrays, the visibility samples lie in a rectangular grid in the frequency domain. When introducing the direction cosines: $x_{n}=R\cos(x_{n})=R\xi$, $y_{n}=R\cos(y_{n})=R\eta$, $z_{n}=R\cos(z_{n})=R\gamma$, Then distance $R_{n}^{c}$ is processed approximately by Taylor expansion of SAIR based on the assumption of far field condition ($R\gg x_{c},y_{c}$) as:

(2) $$R_{n}^{c}=\sqrt{{\left(x_{n}-x_{c}\right)}^{2}+{\left(y_{n}-y_{c}\right)}^{2}+z_{n}^{2}}=\sqrt{{\left(R\xi -x_{c}\right)}^{2}+{\left(R\eta -y_{c}\right)}^{2}+{\left(R\gamma \right)}^{2}}\approx R-(\xi x_{c}+\eta y_{c})$$

The same processing for $R_{n}^{l}$. Therefore, for extended radiation source S, the visibility function in far field is:

(3) $$V_{c,l}=\iint_{\Omega_{s}}T\prime (\xi ,\eta )e^{-j2\pi (\xi u+\eta v)}d\xi d\eta$$

where T′(ξ,η) is a modified target brightness temperature image:

(4) $$T\prime (\xi ,\eta )=\frac{\sqrt{D_{1}D_{2}}F_{c}(\xi ,\eta )F_{l}^{*}(\xi ,\eta )}{4\pi \sqrt{1-\xi^{2}-\eta^{2}}}T(\xi ,\eta )$$

Equation (3) indicates that it has the Fourier relationship between the visibility function and brightness temperature map, on which traditional FFT and G matrix inversion method are established. When the targets are located in near field of the antenna array, the far field parallel approximation adopted in Equation (2) will not hold. So traditional FFT and G matrix inversion methods need to be modified by additional phase compensation in near field [17], where distance $R_{n}^{c}$ and $R_{n}^{l}$ are still processed approximately by Taylor expansion. That also means traditional G matrix inversion method needs to establish different expressions of G matrix in near field and far field.

To reduce multiplicative errors, RRIA adopts an accurate processing of the distance $R_{n}^{c}$ and $R_{n}^{c}$ and modifies the traditional G matrix which is established by the Taylor expansion to establish a new accurate G matrix suitable for both far field and near field [18]. Thus, without being processed approximately by Taylor expansion, Equation (2) can be expressed as:

(5) $$V_{c,l}=\sum_{n=1}^{N}T(n)F_{c}F_{l}^{*}e^{-jK[\sqrt{{\left(x_{n}-X_{c}\right)}^{2}+(y_{n}-Y_{c})+h^{2}}-\sqrt{{\left(x_{n}-X_{l}\right)}^{2}+(y_{n}-Y_{l})+h^{2}}]}$$

Formulate visibility function and brightness temperature images as vectors, and then rewrite Equation (5) in the matrix form directly:

(6) $$V_{M\times 1}=G_{M\times N}T_{N\times 1}$$

where the G(m,n) is the *m-*th row and *n*-th column component of the $G_{M\times N}$

(7) $$G(m,n)=F_{c}(x_{n},y_{n})F_{l}^{*}(x_{n},y_{n})e^{j2\pi \left(\sqrt{{\left(x_{n}-X_{ml}\right)}^{2}+(y_{n}-Y_{ml})+h^{2}}-\sqrt{{\left(x_{n}-X_{mc}\right)}^{2}+(y_{n}-Y_{mc})+h^{2}}\right)/\lambda }$$

Based on the new accurate G matrix, RRIA will reconstruct target brightness temperature images accurately with part of the visibility function.

## 3. The Basic CS Approach Applied to SAIR

This section gives briefly the theoretical fundamentals of CS, and then efficient mathematical formations of the basic CS approach to SAIR are presented.

### 3.1. Principles of CS

Sparse representation has been a powerful approach to image restoration. Suppose a finite signal $x\in R^{N}$ and a much smaller number of observations of x in the form of M linear measurements, we can represent this process mathematically as:

(8) y=Φx

where Φ is an M × N matrix, and $y\in R^{N}$. The matrix Φ represents a dimensionality reduction, where M is typically much smaller than N. Obviously, the system of Equation (8) is underdetermined.

If x is sparse or x has a sparse expansion in a proper basis, it only requires little amount of data to recover x from knowledge of y by solving the *L_0_* norm (the number of its non-zero components) minimization problem:

(9) $$\underset{x\in R^{N}}{\min}{\left\|x\right\|}_{l_{0}},s.t.y=\Phi x$$

where, ${\left\|x\right\|}_{l_{0}}$ is the number of nonzero components in x. However, the recovery needs the combinatorial search which is NP-hard.

CS theory translates this problem into something more tractable by replacing the *L_0_* norm with its convex relaxation *L*_1_ norm. It has been shown that it is very likely to recover x exactly from the *L*_1_-minimization problem provided that x is sparse and that the sensing matrix Φ obeys the RIP condition [4]. If the RIP holds, then the following linear program gives an accurate reconstruction of x:

(10) $$\underset{x\in R^{N}}{\min}{\left\|x\right\|}_{l_{1}},s.t.y=\Phi x$$

where ${\left\|x\right\|}_{l_{1}}=\sum \left|x_{i}\right|$ represents the *L*_1_ norm of x, and $x_{i}$ is the *i*-th component of x. For many natural signals which are not sparse, however, often have concise representations in a convenient basis, *i.e.*, x=Ψθ, where θ is a sparse vector, and then Equation (10) can be reformulated as:

(11) $$\underset{x\in R^{N}}{\min}{\left\|\theta \right\|}_{l_{1}},s.t.y=\Phi \Psi \theta$$

The computation of Equations (10) or (11) is a convex optimization problem, and can be solved efficiently using linear programming methods, which is the foundation for the Basis Pursuit (BP) techniques. There are also a variety of other methods for solving such problems such as Greedy pursuits, iterative thresholding which can be rather fast [19].

### 3.2. The Basic CS Approach to SAIR

To apply the basic CS approach into the SAIR imaging, the random undersampling scheme should be implemented firstly. SAIR images statistics indicate that SAIR images are generally not sparse in the spatial domain but can be sparsely represented in some spaces by transforms such as Discrete Cosine Transform (DCT), wavelet transform. Thus, Based on the traditional G matrix in near field or far field, SAIR imaging framework can be expressed as:

(12) $$V_{M\times 1}=G_{M\times N}T_{N\times 1}=G_{M\times N}D_{N\times N}{T^{\prime }}_{N\times 1}$$

where $D_{N\times N}$ is an orthonormal basis constructed by a single discrete cosine transform (DCT) and the SAIR image has been formulated as a vector.

As RIP requires that the rows of Φ cannot be represented by the columns of Ψ. Designing the matrix Φ of SAIR imaging framework such that the resulting sensing matrix ΦGD has the RIP is a fundamental problem in the basic CS approach. In fact, one can show that the RIP can be achieved with high probability by simply selecting Φ as random matrices, such as Gaussian matrices, Bernoulli matrices, or random partial identity matrices, which are largely incoherent with any fixed basis.

Thus, $\hat{\Phi }$ is constructed by m measurements vectors uniformly randomly selected from sensing matrix Φ which is the identity matrix. $\hat{V}$ is the result of m measurements.

Based on CS theory, reconstruction method of SAIR can be transformed into the following *L*_1_-minimization problem:

(13) $$\min{\left\|T^{\prime }\right\|}_{1}\text{Subject to:}\hat{V}=\hat{\Phi }GDT^{\prime }$$

Obviously, the basic CS approach adopts non-adaptive random measurement matrix $\hat{\Phi }$ and constant basis matrix calculated by DCT transform for SAIR images, and correlation observations of SAIR are nondirective and redundant, thus different features of the unknown scene can be enhanced or weakened which may yield outliers in recovery, so the *L*_1_-minimization solution obtained by the basic CS approach for SAIR imaging is not sparse and robust enough.

## 4. Robust Reweighted *L*_1_-Minimization Imaging Algorithm

In this section, by further using SAIR images statistics, a signal model of robust reweighted *L*_1_-minimization imaging algorithm is obtained in Section 4.1. Then sparse inversion of RRIA is given in Section 4.1.

### 4.1. Signal Model of RRIA

Passive millimeter wave measures the correlation value between pairs of spatially separated antennas receiving the electromagnetic signals from objects in the millimeter wave band, thus considering the multiplicative errors in correlation observations and the receiving noise, according to Equation (12), the signal model of SAIR using the new accurate G matrix established in Section 2 can be written as:

(14) $$\hat{V}=\hat{\Phi }GDT^{\prime }+e$$

where e represents the multiplicative errors in correlation observations and the receiving noise and can be expressed by error functional:

(15) $$\varepsilon^{2}(V:T)={\left\|GT-V\right\|}_{F}^{2}$$

Equation (14) is established on the SAIR images statistics that SAIR images is sparse on DCT basis. To further using SAIR images statistics, prior information estimated by energy functional of SAIR images on DCT basis is expressed as:

(16) $$E^{2}(T\prime )={\left\|T\prime \right\|}_{F}^{2}<E$$

The energy functional of SAIR images gives upper bound of DCT transforms, which constrains outliers in recovery, no matter different features of the unknown SAIR images are enhanced or weakened.

Combining the sparsity and prior information of passive millimeter wave SAIR images in near field, signal model of RRIA using the new accurate G matrix can be written as:

(17) $$\hat{V}=\hat{\Phi }GDT^{\prime }+e,s.t.\{\begin{array}{c} \varepsilon^{2}(V:T)\leq \varepsilon \\ E^{2}(T^{\prime })\leq E \end{array}$$

### 4.2. Sparse Inversion of RRIA

SAIR imaging reconstruction obtains $T^{\prime }$ by inverting Equation (17). RRIA employs reweighted *L*_1_-minimization framework to solve a sequence of *L*_1_-minimization problems, which establishes a more valid optimizing rule by iteratively introducing a weight factor into *L*_1_-minimization problems [20]. Thus, there are two constraint conditions and an iterative weight factor in each *L*_1_-minimization step. By processing the constrained conditions mathematically, RRIA adopts proximal gradient method to solve the each *L*_1_-minimization problem using characteristics of piecewise function by combining the objective function and constrained conditions. The procedure of RRIA can be summarized as the following:

List (1) Set the reweighted iteration count l to 0 and the initial weight W_(0)_ = 1.

List (2) Solve the weighted *L*_1_-minimization:

(18) $${T^{\prime }}_{\left(l\right)}=\arg\min{\left\|W_{\left(l\right)}\times T\prime \right\|}_{1},s.t.\{\begin{array}{c} \varepsilon^{2}(V:T)\leq \varepsilon \\ E^{2}(T^{\prime })\leq E \end{array}$$

RRIA solve the weighted *L*_1_-minimization by solving the closely related problem:

(19) T′(l)=argmin{‖Φ^GDT′-V^‖2+λ1‖W(l)×T′‖1+λ2‖T′‖2}

where the parameter $\lambda_{1},\lambda_{2}>0$ acts as a tradeoff between the sparsity of objective function and constrained conditions. As Φ^G corresponds to a convolutional linear operator characterized by a small-size kernel, RRIA adopts the *k*-th iteration of proximal gradient method to minimize the first term. The second term is inseparable *L*_1_ norm terms, which is convex but unsmooth. It is hard to be minimized directly. The third term is smooth and convex which is remained to be minimized later with the minimization solution of first term.

If the step size t_k_ is selected to satisfy:

(20) tk∈(0,1/‖(Φ^GD)T(Φ^GD)‖

By introducing Equation (20) to formulate a penalty method using an auxiliary variable to decouple the quadratic part from the nonquadratic part, Equation (19) can be easily solved:

(21) $${T\prime }_{k}\underset{T\prime }{=\arg\min}\{f({T^{\prime }}_{k-1})+<(T^{\prime }-{T^{\prime }}_{k-1}),\nabla f({T^{\prime }}_{k-1})>+\frac{1}{2t_{k}}{\left\|T^{\prime }-{T^{\prime }}_{K-1}\right\|}_{2}+\lambda_{1}{\left\|W_{\left(l\right)}\times T\prime \right\|}_{1}+\lambda_{2}{\left\|T\prime \right\|}_{2}\}$$

Thus, by neglecting constant terms and merging quadratic term, Equation (19) becomes:

(22) $${T^{\prime }}_{(l)k}\underset{T^{\prime }}{=\arg\min}\{\frac{1+2\lambda_{2}t_{k}}{2t_{k}}{\left\|T^{\prime }-C_{k}\right\|}_{2}+\lambda_{1}{\left\|W_{\left(l\right)}\times T\prime \right\|}_{1}\}$$

where:

(23) $$C_{k}=\frac{{T^{\prime }}_{k-1}-t_{k}\nabla f({T^{\prime }}_{k-1})}{1+2\lambda_{2}t_{k}}$$

Rewrite Equation (19) from the vector to a set of elements:

(24) $${T^{\prime }}_{(l)k}\underset{T\prime }{=\arg\min}\{\frac{1+2\lambda_{2}t_{k}}{2t_{k}}\sum_{i=1}^{N}{\left(T\prime (i)-C_{k}(i)\right)}^{2}+\lambda_{1}\sum_{i=1}^{N}\left|W_{\left(l\right)}\times T\prime (i)\right|\}$$

Now both the *L*_1_ norm and square of the *L*_2_ norm are separable, *i.e.*, each of them is mere the sum of N nonnegative terms and each of these terms involves only a single variable, the iterate ${T^{\prime }}_{(l)k}$ can be computed exactly by a straightforward shrinkage step as:

(25) $${T^{\prime }}_{(l)k}\underset{T\prime }{=\arg}\sum_{i=1}^{N}\min\{\frac{1+2\lambda_{2}t_{k}}{2t_{k}}{\left(T\prime (i)-C_{k}(i)\right)}^{2}+\lambda_{1}|W_{\left(l\right)}\times T\prime (i)|\}$$

The solution of Equation (25) is:

(26) $${T^{\prime }}_{(l)k}=\sum \max\{|C_{k}|-\frac{W_{\left(l\right)}\lambda_{1}t_{k}}{1+2\lambda_{2}t_{k}},0\}\times sign(C_{k})$$

List (3) utilize the reconstructed ${T^{\prime }}_{\left(l\right)}$ to update the weight factor:

(27) $$W_{\left(l+1\right)}=\frac{1}{{\left\|{T^{\prime }}_{\left(l\right)}\right\|}_{1}}$$

Different from the application and the choice of the weight factor in the [8,18], according to the signal model of RRIA for SAIR imaging, the SAIR imaging system measures the correlation value directly and reconstructs images from the visibility function, which means G matrix is essential for recovery no matter whether SAIR imaging is sparse enough or not. Unweighted *L*_1_-minimization attempts to find the solution with the smallest sum of the magnitudes of nonzero terms. Thus, the framework of unweighted *L*_1_-minimization penalizes larger coefficients more heavily and encourages the growth of smaller ones [11,21]. As using interferometry theory, existence of G matrix limits the number of space sampling points. This is why the SAIR reconstruction based on G matrix often includes pseudo peak and oscillation between the edge of the targets and complex background [22]. To balance the growth of coefficients and guarantee robust and accurate sparsity enhancement, RRIA imposes the new weight factor that is inversely proportional to the magnitude of the SAIR imaging.

List (4) Terminate on convergence or when l attains a specified maximum iteration count *l*_max_. Otherwise, increment l and go to list (2).

RRIA is summarized briefly in Table 1.

**Table 1:** The main simulation parameters of SAIR.

Input	$\hat{\Phi }\hat{,V},K,L,\lambda_{1},\lambda_{2},N$
Algorithm:	Step 1: $k=1,l=0,W\left(l\right)=1,\hat{T^{\prime }}(k-1)=zero(N,1)$;
	Step 2: calculate Φ^GD according to Equation (7);
	Step 3: update $\hat{T^{\prime }}(k)$ by using Equation (26); k=k+1;
	Step 4: if k<K, repeat Step 3;otherwise l=l+1;
	Step 5: if l<L, update W(l) by using Equation (27), set k=1, go to Step 3; otherwise go to Step 6;
	Step 6: calculate T by $T=D^{T}T\prime$
Output	T

## 5. Experiments

In this section, we demonstrate the performance of the RRIA and give the image and numerical comparisons with other algorithm (modified MFFT, G matrix inversion method and the basic CS approach) using the same SAIR imaging system parameters. The MFFT algorithm just needs the visibility function samples for the SAIR imaging system [23]. The G matrix inversion algorithm needs the visibility function samples and the original entire G matrix data which will be used to calculate Moore-Penrose inverse matrices [24]. In Section 5.1, the performance of all methods is evaluated by using the passive millimeter wave target brightness temperature distribution. In Section 5.2, the application of RRIA to real SAIR imaging data is given.

In our comparison, the square SAIR images will be formulated as vectors by all algorithms except modified MFFT. Furthermore, except visual comparisons, the peak signal to noise ratio (PSNR) is introduced for SAIR images quality evaluation as an objective measure defined as follows:

(28) $$PSNR(T^{\prime },T)=10\log_{10}\frac{\max{\left(T\right)}^{2}}{MN\sum_{i=0}^{M}\sum_{j=0}^{N}{\left[T\prime (i,j)-T(i,j)\right]}^{2}}$$

where $T^{\prime }$ is the reconstructed image and T is the original one of size M × N.

### 5.1. Simulation Comparison

As mentioned in Section 2, the simulation model of SAIR imaging adopts a T-shaped antenna array. Square SAIR images (64 × 64) will be formulated as vectors (4096 × 1) by all algorithms except the modified MFFT. The value of images represents the intensity of radiation sources at millimeter wave while Gaussian whiten represents the multiplicative errors in correlation observations and the receiving noise [25].

**Figure 2:**
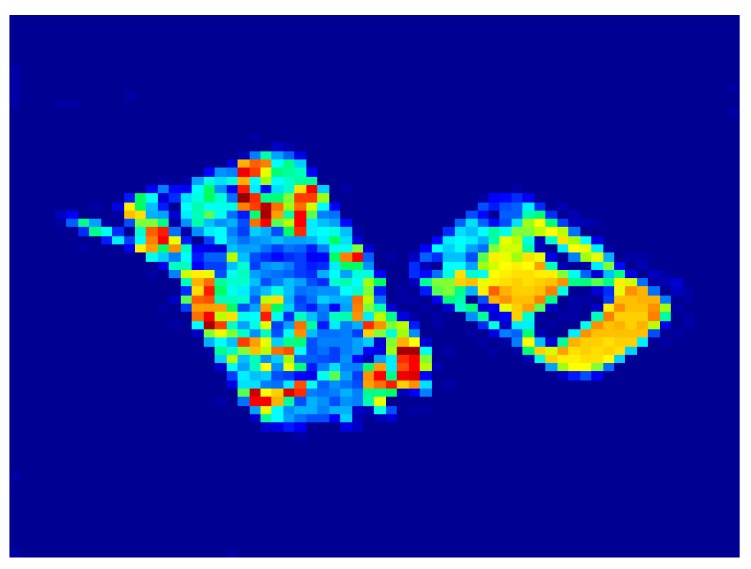
Target brightness temperature distribution of a tank and car.

The SAIR image is presented in Figure 2, where the tank and car body look cooler (brighter) due to cold-sky-reflected radiation of metal in the millimeter wave band. The main simulation parameters of SAIR are listed in Table 2.

**Table 2:** The main simulation parameters of SAIR.

Simulation Parameters	Value
Center frequency	52.8 GHz
Image pixel size	64 × 64
value of image	0~1
Image distance	150 m
Antenna array	140
Entire G size	2500 × 4096
Visibility function samples	50 × 50

Specifically, undersampling of the visibility function also means just the corresponding row vectors of the G matrix need to be calculated based on Equation (7), which also achieves a significant reduction of the data processing.

**Figure 3:**
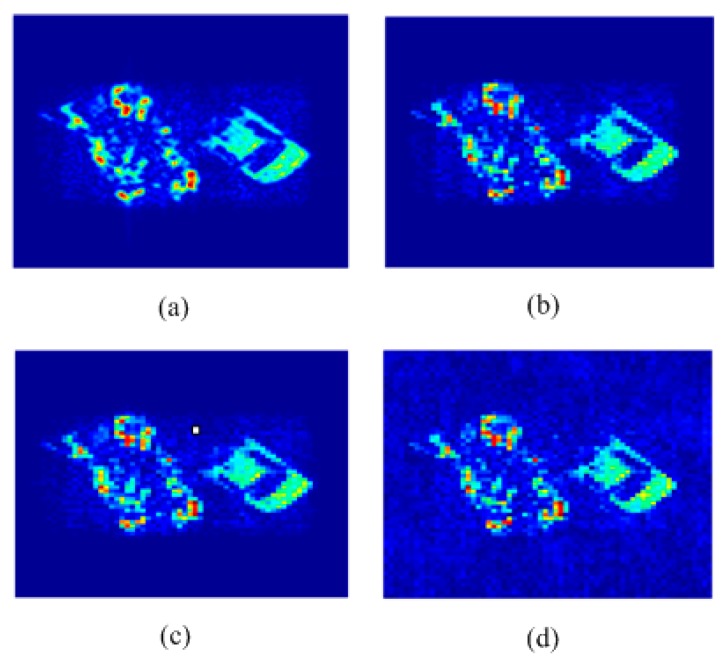
(**a**) Reconstruction image of Figure 2 by RRIA using 70% undersampling; (**b**) Reconstruction image of Figure 2 by CS using 70% undersampling; (**c**) Reconstruction image of Figure 2 by the G matrix inversion method; (**d**) Reconstruction image of Figure 2 by MFFT.

In the first experiments, reconstruction images of Figure 2 by the MFFT and G matrix inversion methods using the entire visibility function samples, the basic CS approach and RRIA using 70% undersampling of visibility function without noise are shown in Figure 3.

As when using interferometry theory, the SAIR imaging system obtains visibility functions directly and the MFFT algorithm just does a Fourier transform for the visibility function samples. From Figure 3d, we can see that MFFT algorithm cannot distinguish the target region and pure background region, even without receiving noise. The pure background region of Figure 3 is influenced by the MFFT algorithm. G matrix inversion method can avoid this problem, but for the tank region which contains many outline details, G matrix inversion reconstruction includes some small pseudo peaks and outliers, which can be seen in Figure 3c. Figure 3b shows that the basic CS approach limits the emergence of these pseudo peaks and outliers. Comparing Figure 3a,b, the pseudo peaks and outliers between the edge of the targets and background are further suppressed. RRIA produces a more visually pleasing result, compared with the results of other methods. A PSNR performance comparison will better illustrate this.

**Figure 4:**
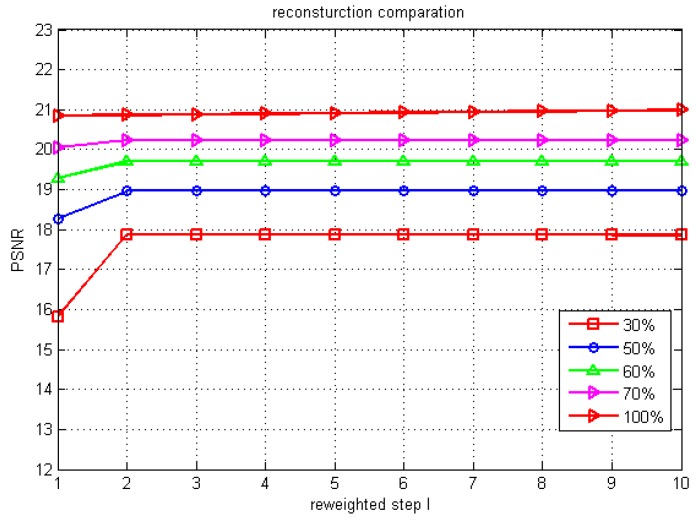
PSNR performances of the reconstruction image of RRIA with 10 reweight steps.

The relationship between the PSNR performance of the reconstructed RRIA images and the iteration count is depicted in Figure 4. The increase of PSNR performance is remarkable during the first two iterations with the increase of degree of undersampling. Specifically, after two iterations, PSNR performance increases 2.05 from 15.81 to 17.86 dB with 30% undersampling. The results presented highlight that unweighted *L*_1_-minimization often cannot get the best PSNR performance in the recovery, whereas reweighted *L*_1_-minimization is capable of improving it efficiently at different undersampling rates for different numbers of iterations. Also, the iteration count could be set to 4 to reduce computation. Note that PSNR performance increases a little with 100% sampling of visibility function, this result which will be analyzed in Section 5.2.

Since the determination of the RRIA parameters is posed as a set of N *L*_1_-minimization problems, the computational cost for computing the RRIA model is O(NSlogS), where S is the size of the sample set used to estimate the RRIA parameters. Thus, compared to the basic CS approach, the increase of the computation time of RRIA mainly depends on reweighted iteration count. As the MFFT algorithm and the G matrix inversion cannot support undersampling, the computation time of the RRIA will be compared to the basic CS approach. When the reweighted iteration count is set to 4, the computation time (Matlab2009b on a PC equipped with eight-core 4GHz AMD processors) of the basic CS approach and RRIA with different undersampling rate of visibility function of Figure 2 is reported in Table 3.

**Table 3:** The computation time of the basic CS approach and RRIA.

Undersampling Rate	50%	60%	70%	80%
t_CS_	21.36s	58.4s	141.5s	371.1s
t_RRIA_	72.7s	210.3s	466.8s	1261.5s

### 5.2. Application to Real SAIR Data

**Figure 5:**
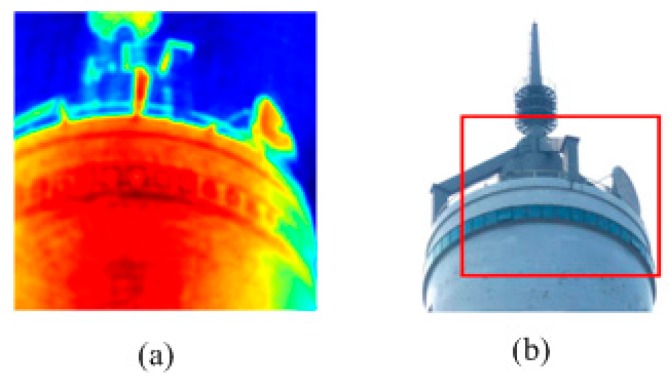
(**a**) Real passive millimeter SAIR image of a tower. (**b**) Visible light image of the tower.

**Figure 6:**
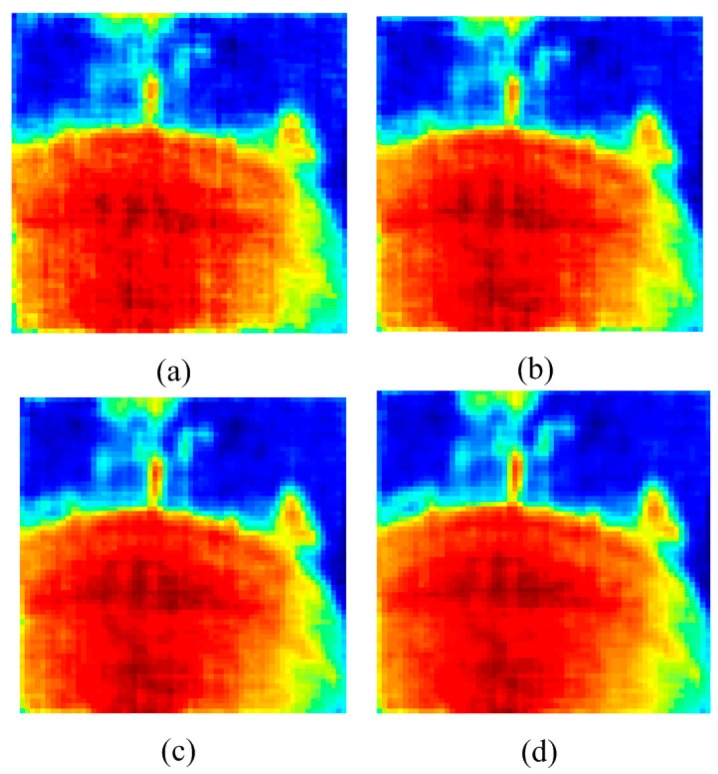
(**a**) Reconstruction image of Figure 5a by RRIA using 40% undersampling; (**b**) Reconstruction image of Figure 5a by RRIA using 50% undersampling; (**c**) Reconstruction image of Figure 5a by RRIA using 60% undersampling; (**d**) Reconstruction image of Figure 5a by RRIA using 70% undersampling.

In this experiment, the RRIA is applied to real SAR data compared to the basic CS approach. The data were acquired by a geostationary SAIR imaging demonstrator in near field by National Space Science Center [26].The test image is shown in Figure 5a while the visible light image of the same scene is presented in Figure 5b. Reconstruction images of Figure 5a by RRIA using 40%, 50%, 60% and 70% undersampling of visibility function with Gaussian noise (variance is 0.02) are presented in Figure 6.

**Figure 7:**
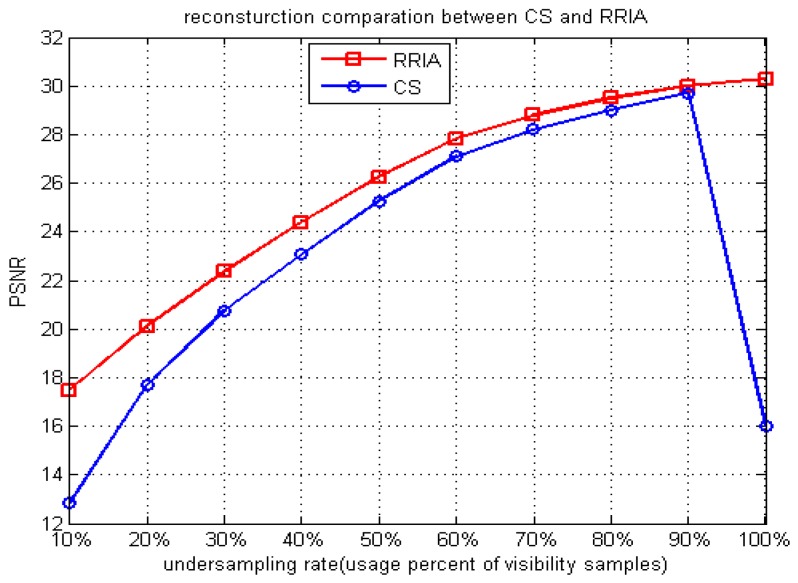
PSNR comparison between CS and RRIA with varying undersampling rates.

In additional experiments, with the same SAIR simulation model, the basic CS approach and RRIA are evaluated at different undersampling rates (usage percent of visibility samples) ranging from 10% to 100% (the step is 10%). Their PSNR performance for the reconstruction images is shown in Figure 7. The PSNR performance of RRIA is better than the basic CS approach at any undersampling rate, but the gap of the PSNR performances decreases as the undersampling rate increases. This indicates that the sparse solution found by the basic CS approach is not correct enough when the undersampling rate is low. In this case, RRIA can improve the solution more effectively. When the undersampling rate is high (using more visibility samples), the basic CS approach could find the better solution so there is little room for improvement by RRIA, which means RRIA is more suitable for low undersampling rates.

While using entire (100%) visibility samples, the basic CS approach has difficulties to find the optimal solution, which demonstrates that the RRIA is robust. We explain it using RIP and the *L*_1_-norm minimization technique. When using entire visibility samples, $\overset{\wedge }{\Phi }$ constructed by m measurements vectors uniformly randomly selected from the identity matrix will not be capable of randomly selecting the vectors from the G matrix. It uses entire G matrix. Thus, the sensing matrix Φ^GD is not random and will not obey RIP. In this case, the basic CS approach could only simply find the solution that minimizes a combination of the data error and the *L*_1_ penalty. It might find the correct solution, but it depends on the penalty more strictly than the case of undersampling while RRIA could solve this problem well, as existing of the constraint condition $E^{2}(T\prime )\leq E$ ensures the validity and robustness of solution of the reweighted *L*_1_-minimization. As mentioned in Section 5.1, while using entire visibility samples, the unweighted *L*_1_-minimization approach can still work very well. That is because $E^{2}(T\prime )\leq E$ is also effective for unweighted *L*_1_-minimization which we have proven in our previous work [27].

**Figure 8:**
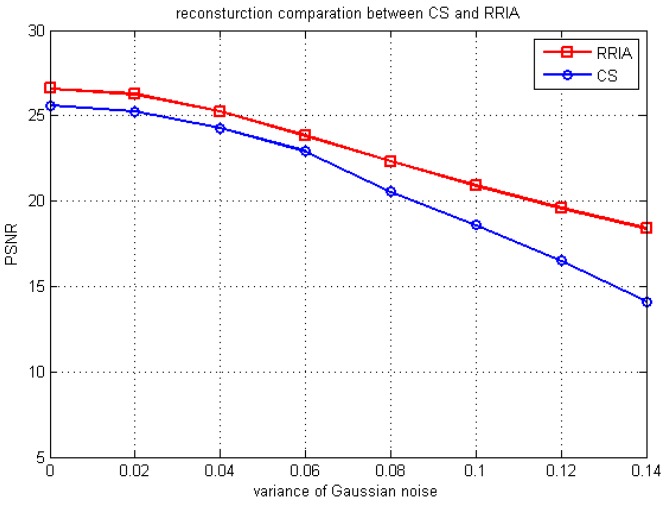
PSNR comparison for CS and RRIA with different variance Gaussian noise.

In practice, due to the multiplicative errors in correlation observations and the receiving noise, SAIR images suffer from noise pollution. It is important to evaluate the robustness of algorithms working under the intense noise interference. The PSNR performance of the basic CS approach and RRIA of reconstruction images of Figure 5a with different variance Gaussian noise is shown in Figure 8, while RRIA and the basic CS approach use 50% of the visibility samples. We can see that the PSNR performance of RRIA is better than the basic CS approach, which means RRIA is more robust and has stronger denoising ability.

## 6. Conclusions

By combining the sparsity and prior information of passive millimeter wave SAIR images in near field, this paper proposes a robust reweighted *L*_1_-minimization imaging algorithm to reconstruct SAIR images accurately. The proposed algorithm employs iterative reweighted *L*_1_-minimization to enhance the sparsity and further uses SAIR image statistics as constraint conditions to make the algorithm more robust for different complex scenes. Experimental results using simulated and real data demonstrate the effectiveness and robustness of the proposed algorithm.
